# Frequency and Clinical Correlates of Thyroid Dysfunction in Patients With Type 2 Diabetes Mellitus: A Cross-Sectional Study

**DOI:** 10.7759/cureus.88962

**Published:** 2025-07-29

**Authors:** Muhammad Zaryab Haider, Muhammad Anees Ur Rehman, Tayyaba Arooj Mufti, Adeel Anwar, Qurat Ul Ain, Rana Arslan Rabbani, Saad Bin Zafar, Muhammad Shoaib Amjad, Amara Sajjad, Zeeshan Rashid Shah, Muhammad Irfan Jamil

**Affiliations:** 1 Cardiology, Rawalpindi Institute of Cardiology, Rawalpindi, PAK; 2 Acute Internal Medicine, Blackpool Teaching Hospitals NHS Foundation Trust, Blackpool, GBR; 3 Internal Medicine, University of Health Sciences Lahore, Lahore, PAK; 4 Cardiology/Medicine, Akhtar Saeed Medical & Dental College, Lahore, PAK; 5 Internal Medicine, University of Health Sciences, Lahore, PAK; 6 Internal Medicine, Worcestershire Acute Hospitals NHS Trust, Redditch, GBR; 7 Medicine, M. Islam Medical and Dental College Gujranwala, Gujranwala, PAK; 8 Internal Medicine, Lahore General Hospital, Lahore, PAK; 9 Medicine, King Edward Medical University, Lahore, PAK; 10 Medicine, East Lancashire Hospitals NHS Trust, Blackburn, GBR; 11 Medicine, Asaaf Hospital, Lahore, PAK; 12 Medicine, Surraya Bukhari Surgical Hospital Mailsi, Mailsi, PAK; 13 Nephrology, Lahore General Hospital, Lahore, PAK

**Keywords:** diabetes mellitus type 2, euthyroid, hyperthyroidism, hypothyroidism, thyroid dysfunction

## Abstract

Background: Thyroid dysfunction commonly coexists with type 2 diabetes mellitus (T2DM), compounding metabolic derangements and increasing the risk of complications. Despite its clinical significance, the prevalence and spectrum of thyroid dysfunction among South Asian patients with T2DM remain incompletely characterized. This study aimed to determine the frequency and types of thyroid dysfunction in patients with T2DM and to examine its associations with demographic characteristics, glycemic control, metabolic parameters, and microvascular complications.

Methods: A cross-sectional study was conducted over a six-month period at a tertiary care hospital in Lahore, Pakistan. Adult patients with confirmed T2DM were enrolled consecutively after informed consent. Clinical assessment included age, gender, body mass index (BMI), duration of diabetes, blood pressure, and glycated hemoglobin (HbA1c), along with documentation of microvascular complications (retinopathy, nephropathy, neuropathy), dyslipidemia, and thyroid profile.

Results: A total of 442 patients were included (mean age 56.12 ± 9.47 years; 228 males, 214 females). Hypothyroidism was present in 105 (23.8%), subclinical hypothyroidism in 66 (14.9%), euthyroid status in 251 (56.8%), and hyperthyroidism in 20 (4.5%). The mean BMI was 26.40 ± 4.33 kg/m², with overweight and obesity most common in hypothyroid (38.1% and 31.4%, respectively) and subclinical hypothyroid (63.6% and 16.7%, respectively) patients. Mean diabetes duration was longest in hypothyroidism (10.37 ± 5.02 years), compared to subclinical hypothyroidism (6.55 ± 5.28 years), euthyroid (5.52 ± 3.69 years), and hyperthyroidism (7.00 ± 4.57 years). Hypothyroid patients reported the poorest glycemic control (mean HbA1c 9.45 ± 1.64%). Microvascular complications (retinopathy 30.5%, nephropathy 20.0%, neuropathy 27.6%) and dyslipidemia (63.8%) were markedly elevated in hypothyroid cases. Statistically significant differences were observed for age, BMI, diabetes duration, and HbA1c across thyroid status groups (all p < 0.001). Thyroid-stimulating hormone (TSH) correlated positively with age (r=0.201), BMI (r=0.131), diabetes duration (r=0.398), and HbA1c (r=0.414).

Conclusion: Thyroid dysfunction, particularly hypothyroidism, was frequent in T2DM and associated with older age, female gender, longer diabetes duration, adverse metabolic profiles, and greater burden of microvascular complications, underscoring the importance of routine thyroid assessment in diabetic care.

## Introduction

Type 2 diabetes mellitus (T2DM) is a leading global health challenge, affecting more than 422 million adults worldwide as of 2014, with prevalence rates continuing to rise due to urbanization, socioeconomic shifts, and lifestyle factors [1]. This burden is most pronounced in low- and middle-income countries, where early-onset T2DM among individuals aged 15-34 years is increasingly common, resulting in accelerated disease progression and a heightened risk of complications [1,2]. In Pakistan, the epidemiological landscape is particularly concerning; recent national surveys estimate that 17.1% of adults have T2DM, representing a 148% increase from prior years. Regional data reveal pooled prevalence rates between 10% and 14% [3,4].

Thyroid disorders are also highly prevalent globally, affecting 5-10% of the general population, with subclinical hypothyroidism constituting the majority of cases [5]. Importantly, thyroid dysfunction is significantly more common among individuals with T2DM than in non-diabetic populations. Studies have consistently reported pooled rates of thyroid dysfunction in T2DM ranging from 13% to over 35% [6,7]. The prevalence of thyroid disorders in the diabetic population is estimated to be roughly double that of the general population, underscoring the clinical significance of this association [5,7].

The interaction between thyroid function and glucose metabolism is multifaceted. Thyroid hormones regulate metabolic pathways central to glucose and lipid homeostasis, modulate insulin secretion, and impact insulin sensitivity. Both overt and subclinical hypothyroidism are associated with increased insulin resistance, as evidenced by higher Homeostatic Model Assessment for Insulin Resistance (HOMA-IR) scores and positive correlations between thyroid-stimulating hormone (TSH) levels and markers of insulin resistance [8,9]. Hyperthyroidism, though less prevalent, also disrupts insulin action and can aggravate glycemic control. Notably, higher TSH and lower free thyroxine (fT4) levels have been linked to poorer glycemic outcomes and worsened lipid profiles among individuals with T2DM [10].

Coexistence of hypothyroidism with T2DM adds meaningful challenges to patient care. People facing both conditions tend to have worse cholesterol levels, especially higher low-density lipoprotein (LDL), and face greater risks of heart disease and microvascular complications like retinopathy, nephropathy, and neuropathy. Managing diabetes becomes more complex, requiring closer follow-up and individualized treatment. The higher likelihood of these issues in women, older adults, those with long-standing or poorly controlled diabetes, and those who are overweight underscores the importance of further regional research to better understand and address thyroid dysfunction in diabetic populations. This study was conducted to determine the frequency and spectrum of thyroid dysfunction in patients with T2DM and to evaluate its associations with demographic variables, metabolic parameters, and microvascular complications in a tertiary care population.

## Materials and methods

This was a cross-sectional study conducted at the Department of Medicine, Lahore General Hospital, Lahore, Pakistan, over a period of one year from October 2023 to March 2024. The study was approved by the Institutional Review Board, Post Graduate Medical Institute/Amer-Ud-Din Medical College /Lahore General Hospital (approval number: IRB0167/08/2023). Informed consent was obtained from all participants.

Participants

Adult patients aged 18 years and above with a confirmed diagnosis of T2DM were eligible for inclusion. The diagnosis of T2DM was made based on documented fasting HbA1c ≥6.5% or medical records. Patients having type I diabetes mellitus, gestational diabetes, a previous history of thyroid surgery, current use of thyroid medications, pregnancy, severe systemic illness, acute diabetic complications, a history of radioactive iodine therapy, pituitary or hypothalamic disease, or use of medications known to affect thyroid function such as amiodarone, lithium, or corticosteroids were excluded. A total of 442 patients who fulfilled the eligibility criteria were included in the study.

Data collection

Baseline demographic and clinical data were collected, including age, gender, BMI, duration of diabetes, glycemic control (HbA1c), comorbid hypertension, and history of microvascular complications (diabetic retinopathy, nephropathy, and neuropathy). All participants underwent venous blood sampling following an overnight fast of 8-12 hours. Laboratory analyses included fasting lipid profile, serum TSH, free triiodothyronine (fT3), free thyroxine (fT4), and HbA1c.

Definitions

Dyslipidemia was defined as the presence of any abnormal lipid parameter based on National Cholesterol Education Program (NCEP) Adult Treatment Panel III (NCEP-ATP III) criteria using enzymatic colorimetric assays on fasting venous samples at baseline [11]. Diabetic retinopathy was defined as typical retinal microvascular changes identified by dilated fundoscopic examination performed by an ophthalmologist at enrolment. Diabetic nephropathy was defined as a urinary albumin-to-creatinine ratio ≥30 mg/g or estimated glomerular filtration rate (eGFR) <60 mL/minute/1.73 m², assessed from fasting morning laboratory samples and confirmed by prior records where available. Diabetic neuropathy was defined as distal symmetrical sensory or motor deficits on clinical examination or abnormal nerve conduction studies, documented by a trained physician at baseline. The classification system defined hypothyroidism as TSH levels exceeding 5.0 mIU/L combined with fT4 below 10 pmol/L, while subclinical hypothyroidism was identified when TSH surpassed 5.0 mIU/L but fT3 (3.1-6.8 pmol/L) and fT4 (10-22 pmol/L) remained within normal ranges. Hyperthyroidism was characterized by TSH below 0.5 mIU/L alongside elevated fT4 and/or fT3 levels, whereas subclinical hyperthyroidism presented with suppressed TSH (<0.5 mIU/L) but normal fT3 and fT4 values. Euthyroid status was assigned when all hormone measurements fell within established reference ranges.

Data analysis

Group comparisons across different thyroid conditions utilized chi-square tests for categorical data and ANOVA for continuous variables. Prior to applying ANOVA, the normality of data distribution was assessed using the Shapiro-Wilk test. When ANOVA indicated significant differences, Tukey's honestly significant difference (HSD) post-hoc analysis was applied to identify specific group differences. Correlation analysis examined relationships between thyroid hormones (TSH, fT3, fT4) and clinical characteristics, including age, BMI, diabetes duration, and HbA1c levels, using Pearson correlation coefficients. Statistical significance was established at p < 0.05. IBM SPSS Statistics for Windows, version 26 (IBM Corp., Armonk, New York, United States) was used for data analysis.

## Results

Among the 442 patients with T2DM, the mean age was 56.12 ± 9.47 years. There were 228 (51.6%) male and 214 (48.4%) female patients. The mean BMI was 26.40 ± 4.33 kg/m². The mean duration of diabetes was 6.89 ± 4.76 years. The mean HbA1c was 8.51 ± 1.53%. Hypertension was present in 129 (29.2%) patients. Diabetic retinopathy was observed in 69 (15.6%), diabetic nephropathy in 60 (13.6%), and diabetic neuropathy in 75 (17.0%) patients. Regarding thyroid status, 105 (23.8%) had hypothyroidism, 66 (14.9%) had subclinical hypothyroidism, 251 (56.8%) were euthyroid, and 20 (4.5%) had hyperthyroidism. Dyslipidemia was identified in 150 (33.9%) patients. The mean TSH level was 7.95 ± 7.83 mU/L (range: 0.1-25.3 mU/L), mean fT3 was 5.08 ± 2.66 pmol/L (range: 1.2-22.1 pmol/L), and mean fT4 was 14.92 ± 7.29 pmol/L (range: 5.1-50.0 pmol/L).

In the hypothyroidism group, most were female (n=71; 67.6%), aged 51-65 years (n=61; 58.1%), and had diabetes for over 10 years (n=55; 52.4%); retinopathy, nephropathy, neuropathy, and dyslipidemia were present in 32 (30.5%), 21 (20.0%), 29 (27.6%), and 67 (63.8%), respectively. Subclinical hypothyroidism was seen more in male patients (n=37; 56.1%), in overweight (n=42; 63.6%), and had neuropathy (n=28; 42.4%). Most euthyroid patients had normal weight (n=120; 47.8%) and lower microvascular complications. In the hyperthyroidism group, all were female, most were in the age group of 35-50 years (n=16; 80.0%), and hypertension was universal. All differences by thyroid status were significant (p < 0.001) (Table 1).

**Table 1:** Comparison of demographic and clinical variables by thyroid status in patients with T2DM (N=442) BMI categories were defined as: underweight (<18.5 kg/m²), normal weight (18.5–24.9 kg/m²), overweight (25.0–29.9 kg/m²), and obesity (≥30 kg/m²). Associations were assessed using Chi-square (χ²) tests. Percentages indicate proportions within each thyroid status category. T2DM, type 2 diabetes mellitus; BMI, body mass index; HbA1c, glycated hemoglobin.

Variable	Category	Hypothyroidism (n=105)	Subclinical Hypothyroidism (n=66)	Euthyroid (n=251)	Hyperthyroidism (n=20)	Chi-square (p-value)
Age group	35–50 years	18 (17.1%)	20 (30.3%)	79 (31.5%)	16 (80.0%)	39.961 (<0.001)
	51–65 years	61 (58.1%)	40 (60.6%)	140 (55.8%)	4 (20.0%)	
	>65 years	26 (24.8%)	6 (9.1%)	32 (12.7%)	0 (0.0%)	
Gender	Male	34 (32.4%)	37 (56.1%)	157 (62.5%)	0 (0.0%)	49.427 (<0.001)
	Female	71 (67.6%)	29 (43.9%)	94 (37.5%)	20 (100.0%)	
BMI category	Underweight	0 (0.0%)	0 (0.0%)	6 (2.4%)	0 (0.0%)	53.993 (<0.001)
	Normal weight	32 (30.5%)	13 (19.7%)	120 (47.8%)	11 (55.0%)	
	Overweight	40 (38.1%)	42 (63.6%)	59 (23.5%)	9 (45.0%)	
	Obesity	33 (31.4%)	11 (16.7%)	66 (26.3%)	0 (0.0%)	
Duration of diabetes	<5 years	12 (11.4%)	36 (54.5%)	120 (47.8%)	6 (30.0%)	82.476 (<0.001)
	5–10 years	38 (36.2%)	13 (19.7%)	99 (39.4%)	8 (40.0%)	
	>10 years	55 (52.4%)	17 (25.8%)	32 (12.7%)	6 (30.0%)	
HbA1c levels	<7%	0 (0.0%)	11 (16.7%)	51 (20.3%)	14 (70.0%)	88.787 (<0.001)
	7.1–8.5%	25 (23.8%)	21 (31.8%)	90 (35.9%)	6 (30.0%)	
	8.6–9%	42 (40.0%)	19 (28.8%)	77 (30.7%)	0 (0.0%)	
	>9%	38 (36.2%)	15 (22.7%)	33 (13.1%)	0 (0.0%)	
Hypertension	Yes	24 (22.9%)	11 (16.7%)	74 (29.5%)	20 (100.0%)	55.577 (<0.001)
	No	81 (77.1%)	55 (83.3%)	177 (70.5%)	0 (0.0%)	
Retinopathy	Yes	32 (30.5%)	16 (24.2%)	19 (7.6%)	2 (10.0%)	34.143 (<0.001)
	No	73 (69.5%)	50 (75.8%)	232 (92.4%)	18 (90.0%)	
Nephropathy	Yes	21 (20.0%)	15 (22.7%)	19 (7.6%)	5 (25.0%)	18.348 (<0.001)
	No	84 (80.0%)	51 (77.3%)	232 (92.4%)	15 (75.0%)	
Neuropathy	Yes	29 (27.6%)	28 (42.4%)	16 (6.4%)	2 (10.0%)	59.493 (<0.001)
	No	76 (72.4%)	38 (57.6%)	235 (93.6%)	18 (90.0%)	
Dyslipidemia	Yes	67 (63.8%)	40 (60.6%)	39 (15.5%)	4 (20.0%)	102.364 (<0.001)
	No	38 (36.2%)	26 (39.4%)	212 (84.5%)	16 (80.0%)	

**Figure 1:**
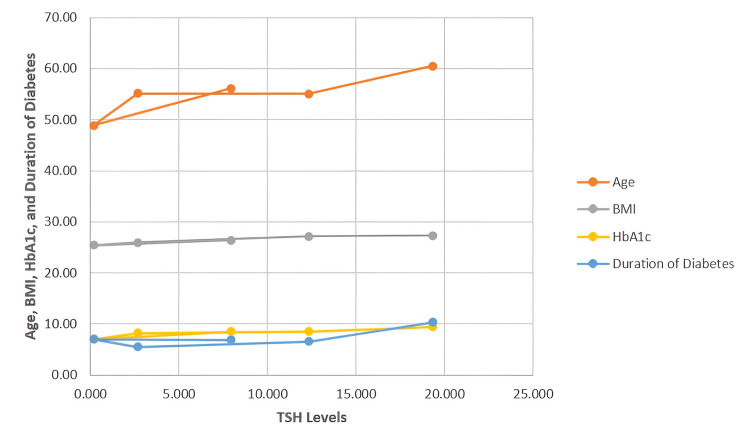
Comparison of age, BMI, HbA1c, and duration of diabetes across TSH levels in patients with T2DM (N=442) T2DM, type II diabetes mellitus; TSH, thyroid-stimulating hormone; BMI, body mass index; HbA1C, glycated hemoglobin

The correlation analysis revealed that TSH exhibited a significant positive association with age (r = 0.201, p < 0.001), BMI (r = 0.131, p = 0.006), HbA1c (r = 0.414, p < 0.001), and duration of diabetes (r = 0.398, p < 0.001), indicating that higher TSH levels were observed among older patients, those with greater adiposity, longer duration of disease, and poorer glycemic control. In contrast, fT3 was inversely correlated with age (r = -0.285, p < 0.001), BMI (r = -0.143, p = 0.003), HbA1c (r = -0.384, p < 0.001), and duration of diabetes (r = -0.202, p < 0.001), suggesting that lower fT3 levels were found in patients with advancing age, higher BMI, worse glycemic indices, and longer disease duration. Similarly, fT4 was negatively correlated with age (r = -0.259, p < 0.001), BMI (r = -0.110, p = 0.021), HbA1c (r = -0.300, p < 0.001), and duration of diabetes (r = -0.181, p < 0.001). Figure 1 is a scatter plot illustrating the relationship between TSH levels (X-axis) and four clinical parameters-age, BMI, HbA1c, and duration of diabetes (Y-axis)-in patients with T2DM. Each parameter is represented by a distinct color and line marker.

Post hoc analysis using the Tukey HSD test demonstrated several statistically significant differences between thyroid status groups for the studied variables. With regard to age, patients with hypothyroidism were significantly older than those with subclinical hypothyroidism (mean difference (MD) = 5.45 years, p = 0.001), euthyroid (MD = 5.32 years, p < 0.001), and hyperthyroidism (MD = 11.58 years, p < 0.001); age was also significantly lower in the hyperthyroid group compared to all other groups (all p < 0.05). Regarding BMI, individuals with hypothyroidism had a higher BMI compared to euthyroid individuals (MD = 1.37 kg/m², p = 0.031), while BMI differences between other thyroid categories were not statistically significant. For duration of diabetes, the hypothyroid group had a significantly longer mean duration compared to subclinical hypothyroidism (MD = 3.83 years, p < 0.001), euthyroid (MD = 4.85 years, p < 0.001), and hyperthyroidism (MD = 3.37 years, p = 0.008); duration was also significantly longer in subclinical hypothyroidism compared to euthyroid (MD = 1.03 years, p < 0.001) and in hyperthyroidism compared to euthyroid (MD = 1.48 years, p = 0.456). For HbA1c, hypothyroid patients had significantly higher levels compared to subclinical hypothyroidism (MD = 0.90%, p < 0.001), euthyroid (MD = 1.23%, p < 0.001), and hyperthyroid (MD = 2.42%, p < 0.001); subclinical hypothyroidism also had higher HbA1c compared to hyperthyroidism (MD = 1.52%, p < 0.001), and euthyroid had higher levels than hyperthyroid (MD = 1.18%, p = 0.002). These findings confirm that hypothyroidism is associated with older age, higher BMI, longer diabetes duration, and poorer glycemic control (Table 2).

**Table 2:** Comparison of age, BMI, duration of diabetes, and HbA1c across thyroid status groups in patients with T2DM (N=442). Values are presented as mean ± SD with 95% CI. One-way ANOVA applied. BMI, body mass index; HbA1c, glycated hemoglobin; SD, standard deviation; CI, confidence interval; T2DM: type 2 diabetes mellitus

Variable	Thyroid Status	Mean ± SD	95% CI for Mean	p-value
Age (years)	Hypothyroidism (n=105)	60.48 ± 8.79	58.77 – 62.18	<0.001
	Subclinical Hypothyroidism (n=66)	55.03 ± 10.82	52.37 – 57.69	
	Euthyroid (n=251)	55.16 ± 9.01	54.04 – 56.28	
	Hyperthyroidism (n=20)	48.90 ± 3.82	47.11 – 50.69	
BMI (kg/m²)	Hypothyroidism (n=105)	27.29 ± 4.44	26.43 – 28.15	0.014
	Subclinical Hypothyroidism (n=66)	27.13 ± 3.60	26.25 – 28.02	
	Euthyroid (n=251)	25.92 ± 4.46	25.36 – 26.47	
	Hyperthyroidism (n=20)	25.40 ± 3.38	23.82 – 26.98	
Duration of diabetes (years)	Hypothyroidism (n=105)	10.37 ± 5.02	9.40 – 11.34	<0.001
	Subclinical Hypothyroidism (n=66)	6.55 ± 5.28	5.25 – 7.84	
	Euthyroid (n=251)	5.52 ± 3.69	5.06 – 5.98	
	Hyperthyroidism (n=20)	7.00 ± 4.57	4.86 – 9.14	
HbA1c (%)	Hypothyroidism (n=105)	9.45 ± 1.64	9.13 – 9.77	<0.001
	Subclinical Hypothyroidism (n=66)	8.55 ± 1.71	8.13 – 8.97	
	Euthyroid (n=251)	8.22 ± 1.25	8.06 – 8.38	
	Hyperthyroidism (n=20)	7.04 ± 0.54	6.78 – 7.29	

## Discussion

The present study identified a substantial burden of both microvascular complications and thyroid dysfunction among patients with T2DM, with clear clinical and metabolic distinctions across thyroid status groups. Regarding thyroid status, hypothyroidism was identified in 23.8% of the patients, subclinical hypothyroidism in 14.9%, euthyroid status in 56.8%, and hyperthyroidism in 4.5%. Notably, patients with hypothyroidism showed the highest mean age, longest diabetes duration, greatest BMI, and poorest glycemic control. These individuals also demonstrated the highest prevalence of microvascular complications and adverse metabolic indices, while euthyroid and hyperthyroid patients showed more favorable profiles.

The prevalence of microvascular complications in this study was 17.0% for neuropathy, 15.6% for retinopathy, and 13.6% for nephropathy, aligning with recent global and regional data reporting neuropathy rates of 10-50%, retinopathy 6-33%, and nephropathy 11-25%. Notably, a multicenter Pakistani survey reported neuropathy in 26.5%, retinopathy in 32.5%, and nephropathy in 21.7% of patients. In another study, the prevalence of microvascular complications was 51.1% for retinopathy, 62.8% for neuropathy, and 32.3% for nephropathy. The comparatively lower complication rates observed in this cohort likely reflect earlier diabetes diagnosis and better glycemic control, as reflected by a mean diabetes duration of 6.89 ± 4.76 years and a mean HbA1c of 8.51 ± 1.53% [12-14].

The frequency of thyroid dysfunction in this diabetic population also aligns with the higher rates described in previous research. Multiple studies have established that thyroid dysfunction is significantly more common in patients with T2DM than in the general population, with pooled prevalence rates of 15-36% and subclinical hypothyroidism as the most frequent abnormality [6,13,15,16]. The current study found hypothyroidism and subclinical hypothyroidism in 23.8% and 14.9% of patients, respectively, mirroring these pooled estimates and matching regional data from South Asia and the Middle East [13,17,18]. Notably, the prevalence of overt hyperthyroidism (4.5%) remains lower, which is consistent with its lesser representation in most international studies [14-16]. These findings strengthen the evidence base for routine thyroid screening in patients with T2DM, especially those with longer disease duration or additional risk factors.

Consistent with previous research, the current analysis identified a strong association between thyroid dysfunction and key demographic and metabolic risk factors. Individuals with hypothyroidism were notably older (mean age 60.5 years), most frequently within the 51-65 year age group, and predominantly female (67.6%), reflecting patterns widely reported in the literature, where both advanced age and female sex increase the risk of thyroid dysfunction in T2DM cohorts [6,19,20]. The study also observed that both hypothyroid and subclinical hypothyroid patients exhibited significantly higher BMI values than euthyroid individuals, in agreement with earlier findings highlighting the contribution of overweight and obesity to thyroid dysfunction in diabetes [6,21]. These associations emphasize the importance of targeted weight management strategies in this clinical context.

Duration of diabetes and glycemic control also emerged as key determinants of thyroid status. The hypothyroid group exhibited the longest mean diabetes duration (10.4 years), significantly exceeding that of subclinical hypothyroid, euthyroid, and hyperthyroid patients. A similar trend has been documented in multiple studies, with longer diabetes duration strongly associated with both subclinical and overt hypothyroidism [6,12,16,22]. Poor glycemic control, as evidenced by higher HbA1c levels, was most pronounced in the hypothyroid group (mean 9.45%), again reflecting established links between thyroid dysfunction and suboptimal metabolic regulation [21-23]. The association between high HbA1c and thyroid dysfunction is bidirectional, with evidence suggesting that hypothyroidism exacerbates insulin resistance and impairs glycemic control, while uncontrolled diabetes may alter thyroid hormone metabolism and sensitivity [8,10].

Correlation analysis provided further insight into these interrelationships. TSH was found to be positively correlated with age, BMI, HbA1c, and diabetes duration, indicating that older, heavier, and poorly controlled patients are at greater risk for elevated TSH and hypothyroid states [24,25]. Conversely, fT3 and fT4 exhibited negative correlations with these same factors, underscoring the progressive decline in thyroid hormone production with increasing metabolic burden and disease chronicity. These findings are consistent with the established pathophysiology of thyroid dysfunction in T2DM, wherein inflammatory, autoimmune, and metabolic mechanisms collectively drive deterioration in thyroid function over time [24-26].

The current results also contribute to the growing literature linking thyroid dysfunction, particularly hypothyroidism, to the development of microvascular complications in T2DM. Hypothyroid patients in this study exhibited substantially higher rates of retinopathy, nephropathy, and neuropathy compared to euthyroid individuals. These findings supported previous literature, where hypothyroidism and subclinical hypothyroidism have been independently associated with increased risk for diabetic microvascular complications [14,22,23,26]. The underlying mechanisms likely involve a combination of endothelial dysfunction, increased oxidative stress, and altered microvascular reactivity in the setting of thyroid hormone deficiency, compounding the adverse effects of chronic hyperglycemia [10,19].

This study highlights the clinical importance of routine thyroid screening in older, overweight, female, or poorly controlled diabetic patients, as early detection may aid in better glycemic control and reduce complications. Its key strengths include a large sample size, thorough clinical and biochemical profiling, and the use of standardized diagnostic criteria. This single-center, cross-sectional study lacked longitudinal follow-up or intervention, limiting generalizability and precluding causal inference between thyroid dysfunction and associated clinical or metabolic parameters. Future research should explore longitudinal outcomes and promote integrated management of diabetes and thyroid disorders to improve long-term care.

## Conclusions

This study demonstrated that thyroid dysfunction was common among patients with T2DM, with hypothyroidism and subclinical hypothyroidism being the most frequently observed abnormalities. Thyroid dysfunction was found to be statistically associated with older age, female gender, increased BMI, longer duration of diabetes, higher HbA1c levels, and a greater prevalence of microvascular complications. These associations suggest that routine assessment of thyroid function may be beneficial in diabetic populations, particularly in patients presenting with high-risk clinical profiles. However, due to the observational and cross-sectional design, no causal relationships can be inferred. Future prospective and interventional studies are needed to explore the potential impact of thyroid dysfunction management on diabetes outcomes.
